# Psychometric evaluation of the child oral impacts on daily performances (C-OIDP) for use in Turkish primary school children: a cross sectional validation study

**DOI:** 10.1186/s12903-020-01162-y

**Published:** 2020-06-19

**Authors:** Kadriye Peker, Ece Eden, Aslı Topaloğlu Ak, Ömer Uysal, Gülçin Bermek

**Affiliations:** 1 Department of Basic Medical Science, Faculty of Dentistry, Istanbul University, Fatih, Istanbul, Turkey; 2 Department of Pedodontics, School of Dentistry, Ege University, Bornova, Izmir, Turkey; 3 Department of Pedodontics, School of Dentistry, Istanbul Aydın University, Küçükçekmece, Istanbul, Turkey; 4 Department of Medical Statistics and Informatics, Medical School, Bezmialem Vakif University, Fatih, Istanbul, Turkey

**Keywords:** Primary school children, C-OIDP, Psychometric evaluation, Factor structure

## Abstract

**Background:**

As patient-reported outcome, the Child Oral Impacts on Daily Performances (C-OIDP) has been commonly used for assessing children’s oral health needs in order to facilitate oral health service planning. It was translated and cross-culturally adapted into Turkish in 2008. Since then, there is no study to assess its psychometric properties in Turkish child population. This cross–sectional study aimed to investigate the psychometric properties and factor structure of the Turkish version of the C-OIDP for use in Turkish primary school children.

**Methods:**

The Turkish translated version was tested on a convenience sample of primary school children aged 11 to 12 years attending two public schools in Istanbul. Data were collected by clinical examinations, face-to-face interviews and self-completed questionnaires. The internal consistency, test–retest reliability, construct validity using exploratory factor analysis (EFA) and confirmatory factor analysis (CFA), criterion related validity (concurrent and discriminant) were evaluated.

**Results:**

A total of 208 children were subjected to the tested the C-OIDP. Overall, 93.7% of them reported at least one oral impact in the last 3 months. The most frequently affected performances were “eating” (72.1%) and “cleaning mouth”, while the performance with the lowest impact was “studying” (13%). The internal consistency and reproducibility of the C-OIDP were acceptable, with a Cronbach’s alpha of 0.73 and an intra-class correlation coefficient of 0.83. The EFA yielded a two-factor model termed “functional limitation” and “psychosocial limitation”. CFA identified the two- factor model which fit the data better than the previously proposed three-factor model, namely physical, psychological and social health. Having malocclusion, the presence of gum disease, reported history of oral problems in the mouth, dissatisfaction with oral health, bad self-rated oral health and having a problem-oriented pattern of dental attendance were found to be the most important factors related to worse oral health- related quality of life, supporting its criterion–related validity.

**Conclusion:**

This study provided preliminary evidence the psychometric properties of the C-OIDP index among Turkish school children aged 11–12 years. It may be applied to evaluate the oral health impact on quality of life in this population.

## Background

Oral diseases and conditions, especially untreated dental caries and associated oral problems affect not only children’s oral functioning but also their general health, family life, social functioning, psychological well-being, and quality of life [1]. Oral health is recognized as being multifaceted and fundamental to overall health and quality of life. It is known that the combined use of oral health–related quality of life measures (OHRQoL), clinical and behavioural indicators in the assessments of populations’ oral health needs provides a more holistic approach for planning oral health services and setting policies which aim to promote children’s their oral health and well-being. Normative methods of assessing dental needs does not reflect non clinical aspects of oral health including its functional and psychosocial aspects. To overcome this shortcoming, OHRQoL measures have been developed to examine the perceived need for dental care and the impact of oral disorders and conditions on individual’s daily life [2, 3].

Unfortunately, there is no practical and validated OHRQoL measure that provides an opportunity to evaluate oral health needs based on their impact and severity on daily performance in the Turkish child and adolescent population [4, 5]. As a socio-dental indicator, the Child Oral Impacts on Daily Performances (C-OIDP) has been commonly used to assess children’s oral health needs in population surveys as well as in clinical studies because of being easier and short [6–9]. This scale was developed and tested in Thai children aged 11–12 years [9] and it was then translated and validated for use in many countries [10–26]. This composite socio-dental indicator is based on the framework of consequences of oral impact which presents a modified version of both the International Classification of Impairments, Disabilities and Handicaps of the World Health Organization and Locker’s model and it focuses on three different levels (the impairment, the intermediate level-pain, discomfort, functional limitation and dissatisfaction with appearance, and the ultimate impacts) in the assessment of oral health consequences [9, 27]. In 2008, this scale was translated and cross-culturally adapted into Turkish through close collaboration with the developers of the C-OIDP at University College London [28]. Until now, no validation study of the translated Turkish version has been conducted in a clinical or population-based sample in Turkish children and adolescents. Before using this measure, its validation should be evaluated in target population [6, 8]. Cultural adaptation process affects the instrument’s content and construct validity at a conceptual level across different cultures. So far, there are a relatively small number of studies examining whether the C-OIDP is unidimensional or multidimensional construct [13, 20, 26, 29], although many psychometric studies have been conducted to validate of this measure in different countries. Due to the widespread international use of the OIDP, measurement invariance that is an important aspect of construct validity representing the same construct across groups within and between populations has to be evaluated in the cross-cultural studies [29, 30].

Therefore, the objectives of this study were to (1) examine the psychometric properties of the Turkish version of the C-OIDP for use in Turkish primary school children and (2) assess its construct validity using both exploratory factor analysis (EFA) and confirmatory factor analysis (CFA).

## Methods

### Participants

The Turkish C-OIDP was tested in a convenience sample of children aged 11 to 12 years attending two public schools located in Fatih Province of Istanbul City during the period March–April 2014. Two public schools where the school oral health promotion program were performed by the Dental Public Health Department of Istanbul University, were selected. One school was in a deprived area and the other in a semi-deprived area. A pilot study involving 52 children was carried out prior to the main study. The sample size was calculated based on a confidence level of 95%, a 5% error and an estimated prevalence of oral impact (87% of children reporting any negative oral health effect) as observed during pilot study. The required sample size was determined to be 174 children. In order to allow a 10% non-response rate, at least 191 subjects should be invited. A total of 232 schoolchildren aged 11–12 years were identified by class lists. Two hundred nineteen parents signed informed consent form for their children participation for this study. Among their children who were included in the study if they were willing to participate in this study, were free from chronic illnesses and disabilities and were present on the day of the survey.

### Procedures

The study protocol was approved by the Ethics Committee of the Faculty of Medicine, the University of Istanbul (register number: 2013/1821). Parent information packs, including the consent form, the information sheet about the study and a brief socio-demographic questionnaire about themselves (e.g., age, education level, health insurance status, monthly family income and number of children in the family) were sent to all parents.

Data were collected through a clinical examination and a structured questionnaires in children. A structured interview schedule consisted of the C-OIDP, self-rated oral and general health, satisfaction with oral health, perceived oral treatment needs, oral health behaviors and socio-demographic information. The first part of the scale was conducted in a small groups of 5 children under the supervison of the the principal investigator (KP). Children selected their perceived oral health problems in last 3 months from a list of 17 oral health problems in the classroom. Children completed individually this oral list, subjective measures to assess general and oral health, their socio-demographic and behavioral characteristics. Then, they completed the second part which assessed oral impacts on 8 daily performances in face-to-face interviews conducted by the principal investigator (KP). If children reported an impact on any performance, the frequency and severity of each reported oral impact were scored. Finally, children with impacts were asked to identify the oral conditions they perceived as causes of their impacts.

### Measures

The C-OIDP index was developed by Gherunpong et al. [9] to assess the oral impacts on eight daily performances: eating food (e.g.,meal, ice-cream); speaking clearly, cleaning your mouth (e.g.,rinsing your mouth, brushing your teeth);relaxing (including sleeping); maintaining your usual emotional state without being irritable; smiling, laughing and showing teeth without embarrassment; carrying out your schoolwork (e.g., going to school, learning in class, doing homework); and contact with people (e.g.,going out with friend, going to a friend’s house). Its first section consisted of self-reported questionnaire on 17 oral health-related problems experienced in the past 3 month that were marked by children. The frequency and severity of each impact were scored on a 3 point Likert scale (Frequency scores: 1- being once or twice a month, 2- three or more times a month, or once or twice a week, and 3- three or more times a week; severity scores: 1- little effect, 2 - moderate effect and 3 - severe effect). Each performance score was obtained by multiplying the frequence score by the severity score. The overall C- ODIP score was is calculated as the sum of the 8 performance scores (ranging from 0 to 72) multiplied by 100 and divided by 72 [9].

#### Turkish adaptation process of the C-OIDP

**Fig. 1:**
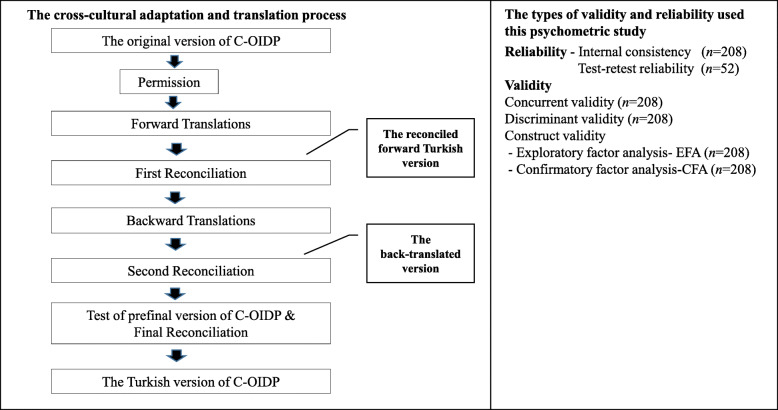
Flow chart describing the psychometric testing and the cross-cultural adaptation

As part of the graduate thesis of Seda Can, the process of translation and cultural adaptation of the C-OIDP into Turkish was carried out in close collaboration with the team developed the original C-OIDP (UCL, London, UK) [28]. After obtaining permission from the developers, this process was performed according to international accepted guidelines [31, 32]. As shown in Fig. 1., this included the following steps:

- Step 1: The C-OIDP was translated from English into Turkish by three dentists and two translators without medical background, whose first language is Turkish.
- Step 2: In the first reconciliation meeting between the forward translators and the Turkish research team, the translated versions of the C-OIDP were reviewed and compared in order to identify any discrepancies, to quarantee cultural sensitivity and to select appropriate wording. The reconciled forward Turkish version was created following discussion and consensus.
- Step 3- It was back- translated to English by two independent native English-speaking professional translators.
- Step 4- In the second meeting, the expert committee composed of research team (three paediatric dentists one of who specialized in OHRQoL assessement) and four translators who examined the source and back-translated questionnaires to achieve semantic, idiomatic, experiential, and conceptual equivalence. The final back-translated version was reached by consensus. The summary report on the difficulties encountered and the making required changes during translation and adaptation process and the translated materials were then sent to the original developers of the C-OIDP at University College London for comparison. A prefinal version of C-OIDP created according to the comments made by original developers and expert panel members.
- Step 5- This prefinal version was then pilot-tested on a convenience sample of 11 children by a resaercher in order to check the application time, applicability and understanding of its questions as well as to simplify its wording. At the end of this step, the reconciled forward Turkish version was obtained according to the comments recorded by children and expert panel. It was then sent to the original developers of the C-OIDP at University College London for approval.

During this process, some words and items were modified according to the comments made by the expert panel and discrepancies in translated questionnaire were resolved. There were minor translation discrepancies in the list of oral health problems. In the list of oral health problems, ‘calculus’ was translated as ‘dental calculus’, because this compound words is commonly used in our country and ‘oral ulcers’ was replaced as ‘sores in the mouth’ in Turkish version because this is more understandable to children. The first question of the second part ‘In the past three months, has any of them caused you any difficulty in (performance)?’ was translated into ‘In the past three months, has any of them caused you any problem in (performance)?. The second question about the severity of the effect was translated as ‘degree of the problem’. In the C-OIDP record form, ‘speaking clearly’ was translated as ‘understandable speaking’ to eliminate its misunderstanding.

Face and content validity were examined by the experts involved in the pilot test to assess the clarity, understandability and simplicity of the items as well as clarity of directions, appropriateness of response categories. According to the expert panel’s judgment and the pretest results, no modifications were made to apply the final Turkish version of the index. Original response options for frequecy and severity of oral impact on daily performance were kept. At this stage, it was also decided not to use the pictures [25].

### Clinical examinations

Following completion of the test-retest study, the children were also clinically examined at school by the same investigator (KP) to assess their dental condition following procedures and diagnostic criteria recommended by the World Health Organization [33]. In this stage, the examiner did not give any information about oral health to the children to prevent changes in their oral health perceptions [8, 10].

The mean number of decayed, missing or filled teeth in the primary plus permanent dentition (dmft + DMFT) was calculated. Students were categorized into those who were caries free (dmft + DMFT = 0) and those with caries experience (DMFT+dmft ≥1) [29, 34]. The gingival index (GI) was used to evaluate the extent of gum disease. Children were categorized into those who had healthy gingiva (GI ≤1) and those with gingivitis (GI > 1) [16]. Malocclusion was assessed according to the Angle’s classification and children were divided into two groups: children with normal occlusion and children with with Angle’s Class I, II, and III malocclusions [35].

### Socio-demographic, behavioural and subjective variables

The following socio-demographic variables were used in the study: child’s gender (male vs female), mother’s age (years) and education level (≤ 8 years vs > 8 years*)* [36]*,* health insurance status (uninsured vs insured), monthly family income (Turkish Lira, TL), and number of children in the family.

The following four questions were included in this survey: satisfaction with oral health (answer ranging from “not at all” to “very satisfied”) [10, 14], perceived dental treatment need (“yes”, “no”) [9, 13, 17], self-rated oral and general health (answer ranging from excellent to poor) [11].

The measure of self- reported oral health behaviors included 4 questions: toothbrushing frequency (≥ twice a day vs ≤ once a day) [37]; use of dental floss (use vs don’t use) [38]; dental attendance patterns (regular dental check-up vs symptom-oriented) [39]; and daily between meals frequency of sugar intake (< three a day vs ≥ three a day) [37].

### Data analysis

The types of validity and reliability used to evaluate the psychometric properties of the Turkish C-OIDP were summarized in Fig. 1. Reliability testing referred to internal consistency and test-retest reliability [40]. The internal consistency of the Turkish C-OIDP was assessed by Cronbach’s alpha, interitem, and item-total correlation coefficients. A Cronbach’s alpha coefficient of a scale above 0.70 and item-total correlation coefficients > 0.20 were regarded as acceptable. An item was considered for removal if its deletion resulted in a > 0.10 increase in the Cronbach’s alpha coefficient. Test-retest reliability was assessed by calculating the intraclass correlation coefficient (ICC) using the two-way random effects model for the C-OIDP score, using the data from the 52 children who were reinterviewed by the same investigator 1 week after the first interview.

The required sample size for test retest reliability was calculated based on the assumptions that the lowest acceptable ICC was 0.8 and the target ICC was 0.9, with a level of significance of 0.05 and a statistical power of 0.80. A minimum sample size of 46 children was needed [41]. Allowing for 10% of losses during follow-up, the sample size was increased to 52 participants.

To establish concurrent validity, the Turkish C-OIDP score was compared between the different groups of subjective oral and general health measures. The underlying hypothesis was that children with favourable perceptions of their oral health were more likely to report lower C-OIDP scores.

Discriminant validity was evaluated by comparing C-OIDP scores of groups that differ regarding the clinical oral health status. The underlying hypothesis was that children with dental diseases would report higher C-OIDP scores (indicating worse OHRQoL) than children free of dental diseases.

The construct validity of the C-OIDP was explored using both EFA and CFA. EFA was performed to identify the underlying dimensions of the C-OIDP. To assess the factorability of the data, Bartlett’s test of sphericity and the Kaiser- Meyer- Olkin (KMO) measure of sampling adequacy were used. Eigenvalues > 1.0, inspection of scree plot, variance > 10% and communality ≥0.30 for each item were used to determine the optimal number of factors [42].

The new structure was then confirmed and compared with existing C-OIDP models by CFA using the following goodness-of-fit indices: Chi-square degree of freedom (Chi-square/df < 5), Comparative Fit Index (CFI > 0.90), Goodness-of-Fit statistic (GFI > 0.90), Normed-fit index (NFI > 0.90), Standardised Root Mean Square Residual (SRMR< 0.08) and Root Mean Square Error of Approximation (RMSEA< 0.10) [43].

To compare directly different models, the Akaike information criterion (AIC), Bayesian information Criterion (BIC) and expected crossvalidation index (ECVI) were used. Within a set of models for the same data, models with the lowest AIC, BIC and ECVI values were regarded as the best fitting model [44, 45].

As the C-OIDP score was not normally distributed, Mann Whitney U test and Spearman rank correlation coefficient were used to analyze the data. To determine the significant predictors of children’s OHRQoL, a backward stepwise multiple linear regression was performed with the overall C-OIDP score as the dependent variable. Socio-demographic characteristics, clinical indices, oral health behaviors, subjective measures were used as independent variables. Variables with *P* < 0.10 in bivariate analyses were entered into the model. For all variables, standardized β coefficients were calculated. In all statistical analyses, the significance level was set to *P* < 0.05. Statistical analysis was performed using IBM SPSS Statistics version 19 for Windows (SPSS Inc., Chicago, IL, USA) and LISREL 9.30 student edition.

## Results

### Sample characteristics

A total of 208 children were subjected to the tested the C-OIDP. A total of 219 children were invited to participate in the validation study, nine children were absent on the clinical examination day and two children refused to participate during the clinical examination (response rate = 95%). There were 55.3% girls and 44.7% boys, with mean age of 11.95 (SD = 0.21). Mean age of mothers was 37.90 (SD = 7.64), 41.3% had formal school education, and 92.3% had health insurance. The mean monthly family income was TL 1497.16 ± 700.65 (or $ US 794).

The mean DMFT was 2.70 (SD = 1.49). 68.8% of children had gingivitis of varying severity, 8.7% had maloclusion, 30.8% visited dentists regularly for check-ups, 54.3% brushed their teeth ≥ twice a day, 18.8% consumed sugar-added products three or more times between meals, and 19.7% used dental floss.

The mean C-OIDP score was 13.11 (SD = 8.71). The most prevalent perceived oral problem was sensitive teeth (48.1%) followed by toothache (45.2%) (Table 1).

**Table 1:** Prevalence of perceived oral problems in the study population (*n* = 208)

List of common oral problems	Children with the problem n (%)
Toothache	45.2 (94)
Sensitive tooth	48.1 (100)
Tooth decay	30.8 (64)
Exfoliating primary tooth	23.6 (49)
Tooth space	2.4 (5)
Fractured permanent tooth	14.9 (31)
Colur of tooth	38.0 (79)
Shape and size of tooth	19.2 (40)
Position of tooth	33.7 (70)
Bleeding gums	36.5 (76)
Swollen gum	17.3 (36)
Dental calculus	4.8 (10)
Sores in the mouth	18.8 (39)
Bad breath	34.6 (72)
Deformity of mouth or face	1.0 (2)
Erupting permanent tooth	9.6 (20)
Missing permanent tooth	1.0 (2)

Overall, 93.7% of the sample reported at least one oral impact in the last 3 months. The performances with the highest frequencies impacts were “eating” (72.1%), “cleaning mouth” (58.7%), and “smiling” (57.7%), while the performance with the lowest impact was “studying” (13%) (Table 2).

**Table 2:** Prevalence of oral impacts on daily performances in the study population (*n* = 208)

**Performances**	**Percentage of children with difficulty carrying out the performance %**
Eating	72.1
Understandable speaking	39.4
Cleaning mouth	58.7
Relaxing	27.4
Emotional status	32.2
Smiling	57.7
Studying	13.0
Contact with people	35.1
At least one of the above	93.7

### Reliability

In terms of internal reliability, the inter-item correlation coefficients among the 8 items of C-OIDP ranged between 0.15 and 0.50 (Table 3). The C-OIDP showed an acceptable reliability (Cronbach’s alpha = 0.72). Alpha value decreased when any item was deleted. Considering item-total correlations, all items were above 0.20 (Table 4). Finally, in terms of test–retest reliability, the ICC was 0.83.

**Table 3:** Correlation coefficients between items

	**Eating**	**Understandable speaking**	**Cleaning mouth**	**Relaxing**	**Smiling**	**Emotional status**	**Studying**	**Contact with people**
**Eating**	1							
**Speaking**	0.313^b^	1						
**Cleaning mouth**	0.501^b^	0.272^b^	1					
**Sleeping**	0.301^b^	0.385^b^	0.284^b^	1				
**Smiling**	0.340^b^	0.323^b^	0.204^b^	0.197^b^	1			
**Emotional status**	0.310^b^	0.260^b^	0.284^b^	0.269^b^	0.173^a^	1		
**Studying**	0.192^b^	0.251^b^	0.208^b^	0.295^b^	0.194^b^	0.123	1	
**Social contact**	0.108	0.237^b^	0.127	0.246^b^	0.198^b^	0.145^a^	0.221^b^	1

^a^Correlation is significant at the 0.05 level (2-tailed); ^b^ Correlation is significant at the 0.01 level (2-tailed)

**Table 4:** Standardised Cronbach’s alpha, item-total correlation and alpha with deleted items

**Performance**	**Corrected item- total correlation values**	**Alpha if item deleted**
Eating	0.56	0.67
Understandable speaking	0.47	0.68
Cleaning mouth	0.49	0.68
Relaxing	0.45	0.69
Smiling	0.39	0.70
Emotional status	0.37	0.70
Studying	0.34	0.71
Contact with people	0.31	0.72

### Concurrent validity

In relation to concurrent validity (Table 5), children with higher C-OIDP scores were less likely to be satisfied with their mouth (*P* < 0.001). C-OIDP scores increased when children reported bad self-rated oral and general health (*P* < 0.001 in both cases). Furthermore, children who perceived a need for dental treatment had higher C-OIDP scores than those who did not have perceived need (*P* < 0.001).

**Table 5:** Findings for concurrent and discriminate validity of the C –OIDP

**Subjective oral and general health measures**	**C-OIDP score Mean (SD)**	***P value***
**Self-rated oral health**^**a**^		
Good (*n* = 155)	11.30 (7.97)	< 0.001
Bad (*n* = 53)	18.39 (8.72)	
**Self-rated general health**^**a**^		
Good (*n* = 137)	10.97 (8.22)	< 0.001
Bad (*n* = 71)	17.25 (8.19)	
**Perceived oral treatment needs**^**a**^		
Yes (*n* = 70)	17.34 (8.07)	< 0.001
No (*n* = 138)	10.97 (8.26)	
**Satisfaction with oral health**^**a**^		
Satisfied (*n* = 119)	10.00 (7.85)	< 0.001
Not satisfied (*n* = 89)	17.28 (8.10)	
**Self-reported oral problems**^**a**^		
Present (*n* = 134)	15.78 (8.07)	< 0.001
Not present (*n* = 74)	8.27 (7.74)	
**Clinical, socio – demographic and behavioral variables**		
**Caries experience**^**a**^		
DMFT+dmft =0 (*n* = 15)	4.81 (7.55)	< 0.001
DMFT+dmft ≥1 (*n* = 193)	13.75 (8.48)	
**Gingival health status**^**a**^		
GI ≤ 1 (*n* = 65)	11.15 (9.02)	< 0.05
GI > 1 (*n* = 143)	14.00 (8.46)	
**Malocclusion**^**a**^		
Present (*n* = 18)	13.94 (8.42)	< 0.001
Not present (*n* = 190)	4.39 (6.95)	
**Child’s gender**^**a**^		
Male (*n* = 93)	12.68 (8.06)	> 0.05
Female (*n* = 115)	13.47 (9.24)	
**Mother’s educational level**^**a**^		
≤ 8 years (*n* = 86)	15.47 (8.15)	< 0.01
> 8 years (*n* = 122)	11.45 (8.75)	
**Tooth brushing**^**a**^		
≥ twice a day (*n* = 113)	12.02 (8.80)	> 0.05
≤ once a day (*n* = 95)	14.41 (8.48)	
**Dental attendance**^**a**^		
Regular dental check-up (*n* = 64)	8.48 (8.15)	< 0.001
Symptom-oriented (*n* = 144)	15.17 (8.17)	
**Dental flossing**^**a**^		
Use (*n* = 41)	9.11 (8.05)	< 0.01
Don’t use (*n* = 167)	14.09 (8.61)	
**Daily between meals frequency of sugar intake**^**a**^		
≥ 3 a day (*n* = 39)	16.59 (9.01)	< 0.05
< 3 a day (*n* = 169)	12.31 (8.47)	
**Health insurance status**^**a**^		
Insured (*n* = 192)	13.31 (8.68)	> 0.05
Uninsured (*n* = 16)	10.67 (8.92)	
**Number of children in the family (r)**	0.273	< 0.01
**Mother’s age (r)**	0.019	> 0.05
**Family monthly income (r)**	0.054	> 0.05

*SD* Standard deviation; *r* Spearman’s rank correlation coefficient. ^a^ Statistical evaluation by Mann–Whitney U-test

### Discriminant validity

With regard to the discriminant validity, children with caries (*P* < 0.001), gingivitis (*P* < 0.05) and malocclusion (*P* < 0.001) had higher C-OIDP scores than their counterparts *(*Table *5**).*

As seen in Table 5, no significant associations were found between the C-OIDP and socio-demographic variables except for mother’s educational level (*P* < 0.01) and number of children in the family (*P* < 0.01). Regular dental attendance pattern, using dental floss, and less frequent sugar intake between meals were associated with lower C–OIDP scores.

The final regression model explained 33.1% of the variance of the overall C-OIDP score (adjusted R^2^ = 0.331, *P* < 0.001). The following factors were identified as predictive of decreased OHRQoL: having malocclusion, the presence of gum disease, having any oral problems, dissatisfaction with oral health, bad self-rated oral health and problem-oriented use of dental services (Table 6).

**Table 6:** Predictors of the overall C-OIDP score in stepwise multiple linear regression analysis

**Variable**	**B**	**SE**	**β**	***P value***
Dental attendance	−2.746	1.182	−0.146	0.021
Self-rated oral health	−2.907	1.250	−0.146	0.021
Satisfaction with oral health	−3.872	1.158	−0.220	0.001
Gingival health status	2.088	1.078	0.111	0.054
Problems in the mouth	3.709	1.172	0.204	0.002
Malocclusion	−5.471	1.854	−0.177	0.004

B, Unstandardized regression coefficient; SE, standard error; β, standardized regression coefficient. Dental attendance: regular dental check-up and symptom-oriented (referent); Self-rated oral health: good and bad (referent); Satisfaction with oral health: satisfied and dissatiesfied (referent); Gingival health status: GI ≤1 and GI > 1 (referent); Problems in the mouth: no problem and some +many problems (referent); Malocclusion: normal occlusion (referent) and malocclusion

### Factorial validity

The KMO was 0.794 and Barlett’s test of sphericity was significant (Chi-square = 267.804, df = 28*;P* < *0.001*) indicating that the data were adequate for the factor analysis. The EFA with varimax rotation yielded a 2-factor solution that accounted for 48.417% of the total variance. The eigenvalue for the first factor was 2.164, explaining 27.056% of the variance and the eigenvalue for the second factor was 1.709, explaining 21.361% of the variance. In addition, an examination of the scree plot confirmed a two-factor structure [35]. Factor 1, named ‘ functional limitation’, consisted of 4 items (eating, understandable speaking speaking, cleaning mouth and smiling; Cronbach’s alpha = 0.67) with factor loading ranging from 0.416 to 0.824. Factor 2 comprised 4 items (sleeping, emotional status, studying and contact with people; Cronbach’s alpha = 0.51) with loadings ranging from 0.558 to 0.748 and was named ‘psychosocial limitation’ (data not shown).

The goodness-of-fit results are demonstrated in Table 7. All models did not indicate the adequate fit to the given data in terms of non-significant chi-square and RMSEA, but CFI, GFI, SRMR, and Chi-square/df were acceptable. For the one-factor model, all fit indices were acceptable except the NFI value which was slightly lower than the cutoff value of 0.90.

**Table 7:** Summary of fit indices of the one- factor model compared to the existing models

	1-factor model	2-factor model^a^	3-factor model^b^
Chi-square (df)	77.14 (20)	73.42 (19)	71.09 (17)
Chi-square degree of freedom	**3.857**	**3.864**	**4.181**
RMSEA	0.117	0.117	0.124
CFI	**0.914**	**0.926**	**0.921**
GFI	**0.925**	**0.928**	**0.925**
NFI	0.889	**0.904**	**0.900**
SRMR	**0.0514**	**0.0528**	**0.0498**
AIC	4997.379	**4806.699**	5408.207
BIC	5050.780	**4870.112**	5464.945
ECVI	0.525	**0.516**	0.524

^a^The suggested new model; ^b^The three- factor model by Mtaya et al. *CFI* Comparative fit index; *GFI* Goodness-of-Fit statistic; *NFI* Normed-fit index; *SRMR* Standardised Root Mean Square Residual; *RMSEA* Root mean square error of approximation; *AIC* Akaike’s information criterion; *ECVI* Expected cross-validation index; *BIC* Bayesian information criterion; acceptable fits in bold

The results of confirmatory factor analysis for all models showed that Chi-square values were statistically significant but RMSEA values were slightly higher than the recommended level. Comparison of CFA on the existing C-OIDP models (three-factor model and one-factor model) and the new two-factor structure in the same sample indicated that the two- and three - factor models yielded acceptable fit indices, with the two -factor model performing slightly better than the three-factor model. Considering the AIC, BIC and ECVI values, the factor structure found in this study provided the best results among the evaluated models [44, 45].

## Discussion

To our best knowledge, this is the first study to assess the psychometric properties of the C-OIDP in a convenience sample of primary school children. Until now, no national oral health survey on the prevalence of oral health impacts of Turkish children using a validated OHRQoL has been conducted in Turkey. Therefore, we choose to use the C-OIDP in this study as it is designed to be incorparated into oral health needs assessment [7–9]. Using this scale in population surveys could help professionals for planning and evaluating oral health promotion activities and oral health services for the community [7–9]. The validity and reliability of measure should be confirmed before quality of life is used as an outcome [46].

The Turkish C-OIDP showed acceptable internal consistency and test-retest reliability. The Cronbach’s alpha was higher than the values reported from previous studies conducted in France [10], England [11], Brazil [12], Peru [13], Spain [14], Italy [15], Chile [18], South India [20] and Moroccan [21]. The ICC was similar to the validation studies in Chile [18], France [10] and South India [20].

The face-to-face interview format was preferred for data collection, because more than half of students aged 11–12 years had difficulty responding to the questions in the second part of the C-OIDP in the pretesting of the Turkish C-OIDP. Similar approach was used in many validation studies [10–13, 15, 16, 18, 20, 22, 29] conducted in similar age groups.

As used in previous studies [10–14, 16–24, 47], we chose to use the weighted OIDP scores in this study, because this scoring method was found to be a better predictor than the unweighted scores (frequence and severity scores) for DMFT [48]*.*

Only four validation studies examined the factor structure of the C-OIDP [13, 20, 22, 28], three used EFA only [13, 20, 26], one used both EFA and CFA [29]. Studies using only EFA [13, 29] suggested a three-factor model consisting of physical, psychological and social health components, whereas Agrawal et al. [20] and Amilani et al. [26] proposed a two factor model which represented the physical and psychosocial health components. Our study followed a similar approach that was used by Mtaya et al. [29] to identify the factor structure of the C-OIDP. We conducted firstly an EFA. The results of the EFA were then tested using CFA on the same sample to obtain an estimate of goodness of fit and to compare the extracted model to the previous model identified in the literature [29]. In our study, a two-dimensional structure was identified with EFA. The first factor reflected the impact of oral conditions on functional limitations while the second factor consisted of items reflecting the psychosocial aspects of OHRQoL. The similar two-factor structure as in Agrawal et al.’s study [20] and Amilani et al.’s study [26] were found. The findings of this study agree with previous studies [13, 20, 29], reporting that the C-OIDP has a multidimensional structure which represent in the theoretical model of oral health consequences [27]. In the study of Mtaya et al. [29], CFA indicated better fit for a three-factor solution than the two- and one-factor model of the C-OIDP. We compared a two-factor solution obtained from the EFA to a three-factor structure [29]. CFA identified the new two- factor model which fit the data better than the previously proposed three-factor model [29]. Our findings based on EFA and CFA suggest that C-OIDP is a multidimentional measure covering functional and psychosocial dimensions. These two dimensions represents the ultimate impacts of oral health consequences. This study provides insight into the underlying factor structure of the total score version of the C-OIDP [49].

Differences in factor structure between studies may be attributed to sampling differences, having various functions and meanings of the C-OIDP items in different cultures, and use of different scoring versions [26, 30, 49] Further study is required to generalise and confirm the two factor structure of the C-OIDP in a large, nationally representative sample of Turkish children.

The prevalence of oral impacts observed in this study is much greater than what was reported in earlier studies in Brazil [12], Thailand [47], France [10], Israel [19]. The most prevalent oral problem reported was sensitive teeth followed by toothache, which were similar to previous studies conducted in Brazil [12], Sudan [16], Italy [15], Israel [19], Malaysia [17], North India [24], and Moroccan [21]. In agreement with most studies [11, 12, 14, 15, 17, 18, 20–22, 29], we found that the performances with the highest frequencies impacts were eating, cleaning mouth and smiling; while the performances with the lowest impact were “studying” and relaxing. This is not surprising, because impacts on functional limitations and psychological well being were more prevalant than impact on social well-being social during children’s transition to adolescence [47, 50]. Similarly, some studies reported that social contact [11, 15, 17–19], emotional stability [14, 29] and speaking [19, 22] were the least frequently reported impacts. These differences in self- reported oral impacts may be related to childrens’ perception about health and ilness which are affected by their stage of development and social context in which they live [47, 51].

Knowledge in existing validation studies is limited, especially in terms of behavioral and socio-demographic predictors of impaired OHRQoL. In previous validation studies using multivariate analysis methods, socio-economic status, child’s age and gender, district of residence, area of residence and type of school were found be important predictors of impaired OHRQoL [15, 16, 20, 29]. In the bivariate analysis, children whose mothers had lower educational level and more children had worse OHRQoL. This may be explained by the fact that these mothers face a number of barriers to accessing oral health care for children and they have lower health literacy, resulting in worse oral health knowledge and their children’s oral health status [5].

Consistent with previous studies used bivariate analysis method, we found that use of dental floss, regular dental check-up [29], and consumption of sugars between meals [16, 29], were associated with worse OHRQoL. So far, only one study reported that the fruit intake frequency and mouthwash were significant behavioral predictors of OHRQoL [15]. In contrast to this study, we found that only routine dental attendance was an important factor of improved OHRQoL.

Consistent with previous studies, we found that perceived need for dental treatment [9–15, 18, 24], dissatisfaction with oral health [9, 11, 12, 14, 24, 29], having oral problems [9, 12, 14, 19, 21, 29], poor perceptions of self-rated general health [12, 29] and oral health [11–13, 16–18, 24, 29] were associated with worse OHRQoL in the bivariate analysis. However, subsequent multivariate analysis showed that SROH, satisfaction with oral health and self- reported oral problems were important subjective factors of OHRQoL. Our findings are consistent with Castro et al. [34], who reported that self-reported oral health problems explained more of the variation in OHRQoL than clinical normative measures.

In some studies, the clinical indices were used in combination with the C-OIDP index [10, 15, 16, 19, 20, 29]. Bivariate analysis revealed that presence of malocclusion, gum disease and dental caries were significantly associated with children’s OHRQoL. Only malocclusion was found to be a significant predictor of impaired OHRQoL of Turkish children in the multivariate analysis, which is consistent with previous studies employing multivariable analysis [15, 20].

This study has some limitations and strengths that must be taken into account when interpreting its results. This study provided initial support for the reliability and validity of the Turkish C-OIDP in a convenience sample of primary school children aged 11–12 years from two public schools in Istanbul. Thus, our findings could not be generalized to the population of interest. This study was conducted on students at two primary public schools, one of that located in a deprived area but others in a semi-deprived area. This could lead to bias because children from families living in poverty and deprivation are more likely to have oral diseases, resulting in reduced children’s and parents’ quality of life [1]. Future representative population-based studies are needed to understand the impacts of family socio-economic status and school-related factors on children’s OHRQoL. Due to cross-sectional design, this study did not verify any cause-effect relationship among the assessed variables and any changes in scores over time. Population-based cohort studies and further validation study in children of different age groups are needed. Future study using the Item Response Theory may provide additional information to the Classical Test Theory and allow performance assessment of individual items [8, 49]. Additional research aiming to compare psychometrically different administration modes of the Turkish C-OIDP index may be useful for selection the effective data collection mode in future epidemiological studies of child populations as well as in clinical settings [52, 53]. We did not use the intensity and extent of impacts as an alternative method of reporting the severity of oral impacts. Future studies using this scoring method may provide useful information on the extent and severity of oral health conditions in the target population when planning and evaluating oral health programmes and services [54]. Additional study using the condition-specific specific C- OIDP may provide an oppurtunity to discrimate between groups with different levels of normative treatment needs in children when planning oral heath services [55].

The CFA and EFA conducted on the same data set in this study. This approach are accepted as less informative [56]. Therefore, further research using CFA is necessary to investigate whether the factor structure can be replicated in the new dataset. The factorial structure of the OIDP in the total score version was examined in this study. Future study on understanding the cross-cultural differences in factor loadings and the impacts of use different scoring methods may provide additional insight into the interpretation of the OIDP factor structure [30, 49].

The main strengths of this study are that multivariate analysis was used to analyze the factors of OHRQoL and the factor structure of the C-OIDP was evaluated through both EFA and CFA methods. This study may provide a compherensive evaluation of the clinical, behavioural, socio-demographic and subjective factors that influence OHRQoL. Furthermore, the findings from this study revealed additional insights into the factor structure of the C-OIDP in the total score version*.*

## Conclusions

This study provided preliminary evidence concerning validity and reliability of the Turkish C-OIDP among primary school children aged 11–12 years. Future studies should be conducted to evaluate its psychometric properties in a population based studies among children and adolescents. Using this scale may provide the opportunity for oral health professionals to identify subgroups of children at risk of poor oral health when conducting national oral health survey as well as for comparing similarities and differences in oral impacts among children in different countries.

## Data Availability

The datasets used and/or analysed during the current study are available from the corresponding author on reasonable request.

## Abbreviations

C-OIDP: Child Oral Impacts on Daily Performances Index
EFA: Exploratory factor analysis
CFA: Confirmatory factor analysis
OHRQoL: Oral health quality of life measures
dmft + DMFT: The mean number of decayed, missing or filled teeth in the primary plus permanent dentition
GI: The gingival index
TL: Turkish Lira
ICC: The intraclass correlation coefficient
KMO: The Kaiser- Meyer- Olkin measure
Chi-square: Chi-square degree of freedom
CFI: Comparative Fit Index
GFI: Goodness-of-Fit statistic
NFI: Normed-fit index
SRMR: Standardised Root Mean Square Residual
RMSEA: Root Mean Square Error of Approximation
AIC: Akaike information criterion
BIC: Bayesian information Criterion
ECVI: Expected crossvalidation index
